# An assessment of outcomes with intramedullary fixation of fractured ribs

**DOI:** 10.1186/s13019-016-0510-3

**Published:** 2016-08-05

**Authors:** Silvana Marasco, Margaret Quayle, Robyn Summerhayes, Ilija D. Šutalo, Petar Liovic

**Affiliations:** 1 Cardiothoracic Surgery Department, the Alfred Hospital, 55 Commercial Rd, Prahran, 3181 Australia; 2 Department of Surgery, Monash University, Melbourne, VIC Australia; 3 Faculty of Science, Monash University, Melbourne, VIC Australia; 4 Commonwealth Scientific and Industrial Research Organisation (CSIRO), Mathematics Informatics and Statistics, Melbourne, VIC Australia

**Keywords:** Rib fracture, Rib fixation, Intramedullary, FEA modelling

## Abstract

**Background:**

Surgical management of fractured ribs with internal fixation is an increasingly accepted therapy. Concurrently, specific rib fixation prostheses are being developed which should improve results and minimise hardware and rib/splint construct failures. The Synthes titanium intramedullary splint lends itself to difficult to access areas such as posterior rib fractures and fractures under the scapula. We analyse a case series of patients in whom this rib fixation prosthesis has been used.

**Methods:**

Fifteen patients received 35 intramedullary splints. Follow up at 3 and 6 months was performed with three dimensional computed tomography scanning to assess for bone alignment, callus formation and healing, residual deformity, hardware failure or cut through. Computerized finite element analysis (FEA) was used to model forces acting on a posterior fracture with and without an intramedullary fixation splint in situ.

**Results:**

Complete healing (bony union) was noted in only 3 (9 %) of the fractures fixed with splints by 3 months. Partial healing (cartilaginous union) was noted in 28 of the 33 fractures (85 %), and non healing was noted in only 2 (6 %). In both those two patients, failure at the rib / splint interface was noted after both patients reported sneezing. No hardware failures were noted. By 6 months the fractures which had shown partial healing, had all completely healed. There were no late failures (between 3 and 6 months) of either hardware or rib/splint interfaces.

FEA modelling identified sites of increased stress in the rib at the rib / splint interface and in a modelled intramedullary splint where it spans the fracture.

**Conclusions:**

Further analysis of outcomes with intramedullary splints is warranted as well as further development of intramedullary rib fixation solutions.

## Background

Surgical management of fractured ribs with internal fixation is becoming an increasingly accepted therapy particularly in patients with flail chest injury. Benefits include shorter ventilation times, earlier discharge from the intensive care unit, cost savings, and earlier return to work [1–3]. Concurrently with an increased interest in rib fixation, specific prostheses have become available. Until recently, lack of specific rib fixation prostheses led to the use of alternatives such as K wires, cerclage sutures, and off label absorbable prostheses which have been associated with hardware failures, perforation or migration [4, 5]. The development and availability of specific rib fixation prostheses in recent years such as the MatrixRib (DePuy Synthes, West Chester, PA, USA), RibLoc (Acute Innovations, Hillsboro, OR) and Stratos (Strasbourg Thoracic Osteosyntheses System - STRATOS™; MedXpert GmbH, Heitersheim, Germany) has addressed this deficiency. All three of these systems rely on outer cortical placement of plates with either bicortical screws or malleable ‘claws’ to attach the titanium prosthesis to the rib [6–8]. However, rib fractures underneath the scapula are particularly problematic as it is almost impossible to ensure that drill holes and screws are placed perfectly perpendicular to the plates in this area. Posterior fractures are also problematic, given their position and muscle layers overlying the posterior ribs, and the increased propensity to hardware failure in this position [4, 9].

**Fig. 1:**
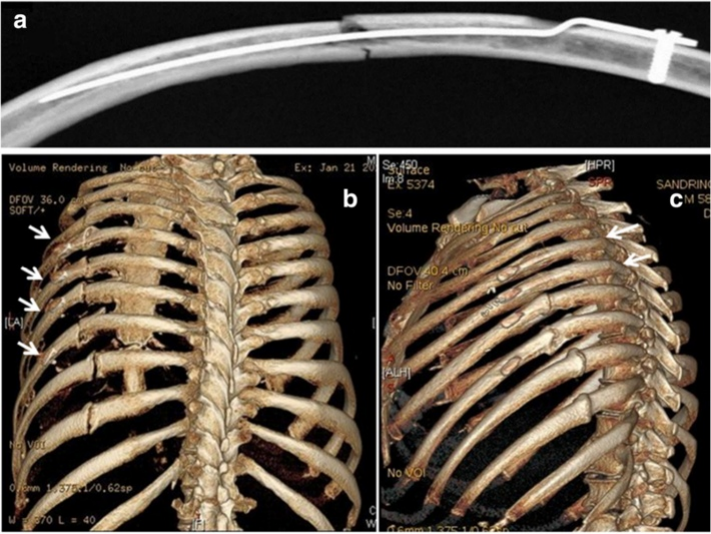
**a**. Intramedullary splint in situ [10, 11]. **b**. Splints used to fix fractures in left ribs 5–8. 3D CT scan at 3 months shows good alignment but little bony bridging across the fracture site (except for the fifth rib – most superior rib fixed). In fact the amount of bony callus formation is comparable to the 9^th^ and 10^th^ rib fracture sites which were treated conservatively. **c**. Same patient at 6 months with complete healing of all ribs. The fracture sites in ribs 5 and 6 are imperceptible

The Synthes intramedullary rib splint has been developed as a rib fixation option that overcomes the biomechanical limitations of Kirschner wires. Although recommended as an option for single rib fractures, the splint does lend itself to the more difficult to access rib fractures. The prosthesis itself is a 1 mm thick titanium splint that is available in 3,4 or 5 mm widths depending on the rib size. The splint is inserted via an outer cortical drill hole of 5 mm, located approximately 30 mm proximal to the fracture. The 97 mm long splint is then ‘hammered’ along the intramedullary canal, spanning the fracture [10]. The proximal part of the splint is contoured to sit flush on the outer cortex and is fixed into position with a single bicortical screw. There is no distal fixation as such, the stabilisation of the rib fracture being achieved by the stiffness of the splint within the intramedullary canal (Fig. 1a).

We have utilised the Synthes rib splint to address difficult to access rib fractures, usually in combination with the Synthes Matrix Rib plates. However the lack of distal fixation in the splints stimulated us to critically appraise the healing of the ribs fixed with the splints and to further investigate the behaviour of intramedullary fixation options using finite element analysis (FEA). Our concern was that the splint, although keeping the fractured rib in alignment, was not able to counteract distraction forces on the fracture and thus may lead to suboptimal healing.

## Methods

Seventy three consecutive patients who underwent rib fixation surgery at The Alfred Hospital, Melbourne, Australia between October 2012 and April 2015 were reviewed. Alfred Hospital Institutional ethics approval was gained (HREC # 399/11) and the requirement for individual patient consent was waived.

The Synthes Matrix Rib outer cortical plates were used exclusively in 58 patients and are our standard method for rib fixation. The remaining fifteen patients received 35 intramedullary splints and are the basis of this study. Of those 15 patients, nine also received Synthes Matrix Rib outer cortical plates. In six of those patients the outer cortical plates were for contralateral fractures and in the remaining three, a combination of outer cortical plates and intramedullary splints were used in consecutive ipsilateral fractures. One patient had fractures fixed with Acute Innovations Ribloc outer cortical U plates and presented with hardware movement requiring replacement of one plate with an intramedullary splint.

All patients had multiple fractured ribs and required fixation of more than one rib. The decision to use an intramedullary splint was based on difficulty of access with the outer cortical plates. Thus the intramedullary splints were used almost exclusively for fractures under the scapula. Preoperative imaging with three dimensional computed tomography (3D CT) was performed in all patients to confirm the diagnosis and plan the surgery. Follow up at 3 and 6 months was performed where possible with further 3D CT chest at both time points.

Post-operative 3D CTs were assessed for bone alignment, callus formation and healing, residual deformity, hardware failure or cut through. Complete healing on CT was defined as continuity of the bony cortex with a barely visible fracture line. Partial healing was defined as good alignment with evidence of callus but a clearly visible fracture line remaining. Non-union was defined as failure of alignment and no bridging callus at the fracture site.

The definition of the location of the fractures into posterior, lateral and anterior were defined by the posterior and anterior axillary lines, whereby the posterior axillary line defines the border between posterior and lateral fractures, and the anterior axillary line defines the border between lateral and anterior fractures.

Surgical technique has been previously described [3, 12]. In brief, only rib fractures between the levels of ribs 3 and 10 were fixed, usually requiring only one or two incisions. Choice of rib prosthesis was according to surgeon discretion but generally the rib splints were used for any fractures considered relatively inaccessible for a standard outer cortical plating approach. Specifically, fractures of ribs under the scapula, and posterior fractures (particularly when the patient was in a lateral position on the operating table) were addressed with rib splints. Wherever possible, chest wall muscles were preserved, splitting them along the length of their fibres. The periosteum was also preserved. As it is relatively easy to perforate the thin rib cortex with the rib splints, intraoperative mobile radiological imaging was performed in all cases to ensure that the distal end of the splint had remained within the medullary canal. All operations were performed by the cardiothoracic unit. With institutional ethics approval, the requirement for individual patient consent was waived.

### Finite Element Modelling (FEA)

FEA was utilised to model the forces acting on a posterior fracture with and without an intramedullary fixation splint in situ. The model used has been previously described [8, 12] and incorporates all muscle forces acting on the rib as well as the intrathoracic pressures generated during breathing and coughing. The ANSYS 13.0 package (ANSYS 2011) was used for the solid mechanics FEA. The model aims to identify the likelihood and direction of movement at the fracture site with the intramedullary fixation in situ. Failure is determined by the likelihood of the ultimate tensile strength (UTS) of either the rib cortical bone or the intramedullary splint being exceeded. Various intramedullary splints were modelled in this analysis, although the Synthes titanium splint was not itself specifically modelled.

## Results

Thirteen of the fifteen patients underwent rib fixation within seven days of their injury. The remaining two patients presented with chronic pain and malunion (one also with hardware pain where the Ribloc plate was impinging on the scapula) and were treated between 6 and 12 months after their injury. One patient (female aged 77 years), died within the same hospital stay from other injuries, having had two intramedullary splints inserted. As there is no follow up imaging on this patient, she has been excluded from further analysis. The remaining 14 patients were all male. The average age of the 14 patients was 52 years (range 32–77 years). Six of the 14 patients had a flail segment as part of their rib fracture injury. The median number of fractured ribs per patient was 7 (range 4–14). The median number of ribs fixed per patient was 5 (range 4–7) with a median of 2 splints (range 1–5) used per patient as part of the fixation strategy. Thus there were 33 fractures fixed with intramedullary splints available for follow up and assessment.

Of the 33 intramedullary splints placed, six were placed laterally and the remaining 27 splints were used for posterior rib fractures. The direction of the splint insertion (either directed posteriorly toward the spine or laterally away from the spine) was up to surgeon preference. The operating room time was slightly longer than with the use of only outer cortical plates because we use intra operative image intensifier to ensure correct placement of all intramedullary splints. On two occasions, the intramedullary splint had perforated the rib cortex and needed to be backed out and redirected correctly. We have not presented data on operative times here as often these operations were shared with other specialists attending to other (non rib) fractures.

The 3 month CT scans were assessed for degree of healing and presence of residual deformity at the fracture fixation site. Complete healing (bony union) was noted in only 3 (9 %) of the fractures fixed with splints by 3 months. Partial healing (cartilaginous union) was noted in 28 of the 33 fractures (85 %), and non-healing was noted in only 2 (6 %). In both those two patients, there was a failure at the rib/splint interface with removal of the hardware prior to the 3 months, residual deformity and failure of union (shown in Figs. 3 and 4). In no other patient was residual deformity or non-union noted at a fracture site fixed with an intramedullary splint. There were no hardware failures in this cohort.

Pain was markedly reduced at 3 months in all patients with only two patients still requiring opiate analgesics. 10 of the 14 patients had been working prior to their accident and seven of the 10 had returned to work by 3 months. Of the three patients who had not returned to work, one reported ongoing thoracic neuralgic pain associated with the site of rib fractures. Two patients had other injuries which had impacted on their ability to return to work. The remaining four non working patients reported no limitations to their acts of daily living attributable to their chest wall pathology. By 6 months the fractures which had shown partial healing, had all completely healed. There were no late failures (between 3 and 6 months) of either hardware or rib/splint interfaces.

**Fig. 2:**
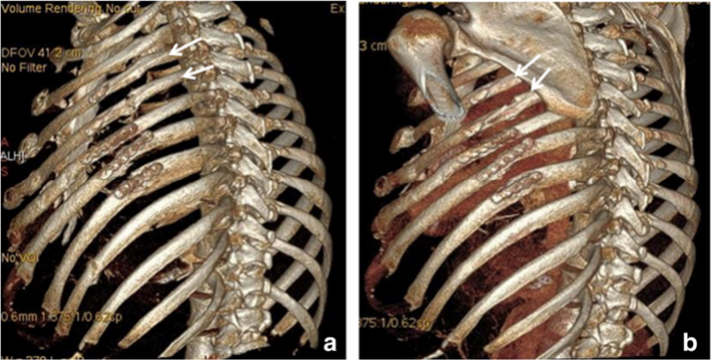
**a**. 48 year old man with intramedullary splints in ribs 4 and 5 with cortical plates on the ribs below at 3 months post operatively. Rib 4 has healed completely with rib 5 showing bridging callus. **b**. Same patient at 6 months with complete resolution of pain at the fracture sites

Figure 1b shows a typical finding at 3 months with good alignment but only partial healing at the fracture sites. By 6 months healing was complete (Fig. 1c). Similar findings demonstrated in the rib fracture in Fig. 2.

**Fig. 3:**
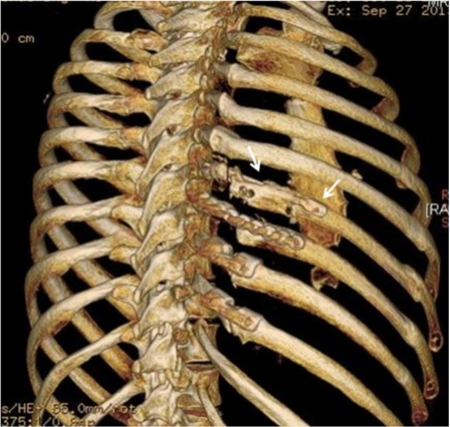
Intramedullary splint in the 7^th^ rib with movement of the lateral fracture segment inferiorly and cut through of the intramedullary splint through the superior cortex. The splint was removed and the fracture site excised with good resolution of symptoms. This patient underwent delayed rib fixation and it can be seen that all of the fixed ribs are not perfectly aligned with inferior displacement of the lateral rib fracture end

Three patients had splint cut throughs which all occurred through the superior cortex. One patient was asymptomatic (Fig. 1b, ribs 7 and 8 exhibit a degree of cut through which was not noted on the initial post operative chest Xray). Two patients were symptomatic requiring splint removal. In both these patients the splint cut through was thought to occur after a sneeze, after which both patients reported a sudden and marked increase in pain in the area. One of the patients had been operated on for chronic malunion and had the splint inserted at a second operation because the existing Ribloc plate was causing clicking against the scapula. The Ribloc plate was removed at the second operation and replaced with the Synthes splint (Fig. 3).

**Fig. 4:**
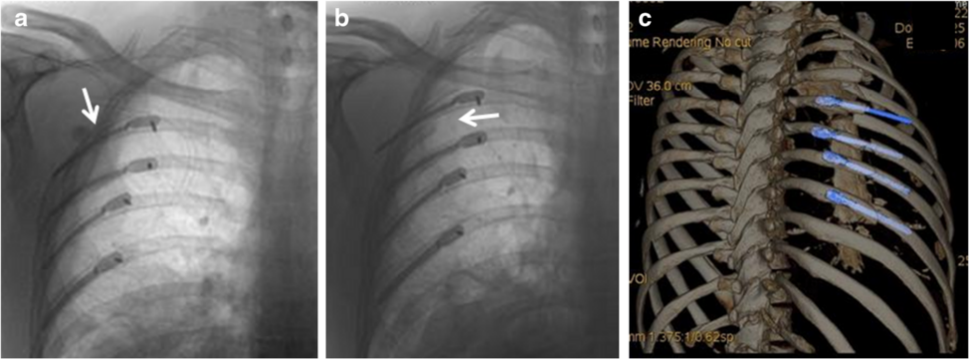
**a**. Showing good intramedullary position of the uppermost rib splint. **b**, **c** The distal part of the uppermost rib splint can now be clearly seen to be outside the medullary canal. The distal rib segment has displaced inferiorly and posteriorly with the proximal (posterior segment) now overlapping

The second patient had four splints placed via a posterior approach with intraoperative imaging by fluoroscopy and a postoperative chest Xray confirming good position (Fig. 4a).

However, on day 3 post operatively the imaging had dramatically changed necessitating removal of the uppermost splint (Fig. 4b, c).

### Finite element analysis

**Fig. 5:**
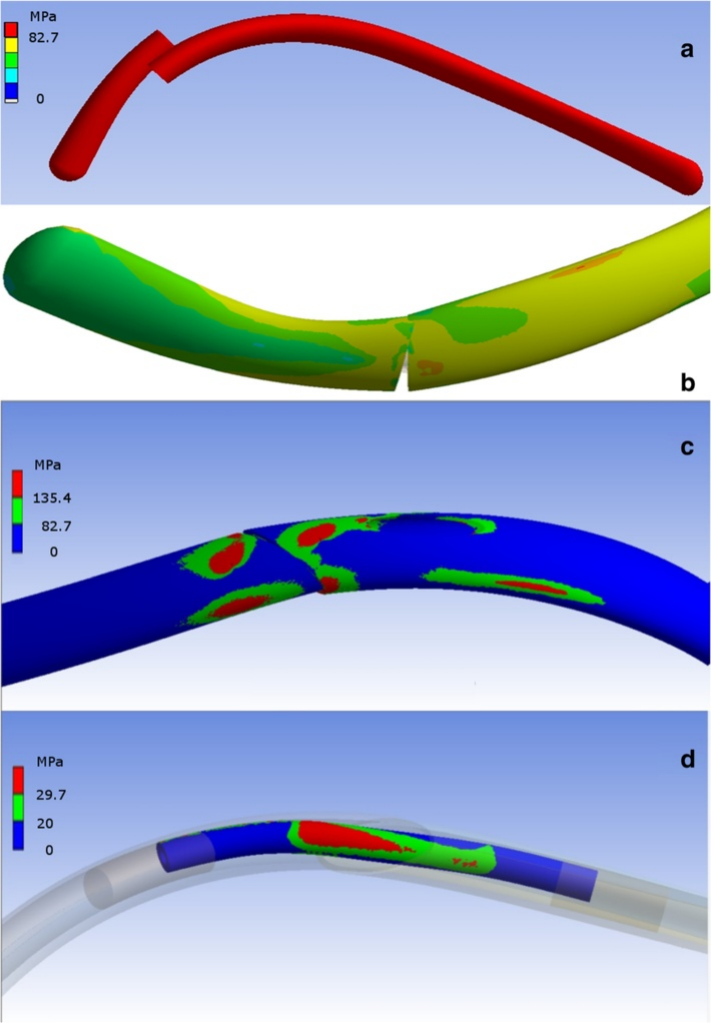
**a**. FEA modelling of a sixth rib with posterior fracture subjected to all attached muscle forces and physiological intrathoracic pressures. **b**. FEA modelling of posterior rib fracture with an intramedullary splint in situ at normal breathing intensity forces. Movement is noted at the fracture site but the ultimate tensile strength (UTS) of the bone at the fracture site and the UTS of the intramedullary splint are not exceeded in this modelling run. **c**. Assessment of a spiked steel intramedullary splint shows the UTS of the cortical bone is likely to be exceeded at those points where the spikes are in contact with cortical bone during coughing intensity forces. During normal breathing simulated forces, high stresses were not observed. **d**. An idealised intramedullary splint of bioresorbable polymer showing that the UTS of the splint itself is being exceeded at the fracture site

Modelling of a posterior fracture in an idealised sixth rib confirmed overlap of the fracture ends with repetitive typical forces acting on that rib [8, 12, 13] (Fig. 5a). This mirrors the displacement seen clinically as demonstrated in Figs. 3 and 4.

Assessment of movement occurring at the posterior fracture site with an intramedullary splint in situ confirmed some degree of movement continuing to occur (Fig. 5b). Assessment of a spiked steel intramedullary splint spanning a posterior fracture showed the UTS of the cortical bone was likely to be exceeded where the splint contacted the bone when forces were increased to cough strength forces (Fig. 5c). Both rib-splint construct failures noted in this series occurred after the patient reported sneezing. Sneezing generates a higher intrathoracic pressure than coughing (±60 kPa compared to ±40 kPa). With regards to the intramedullary splint itself, any intramedullary splint is placed under greatest strain where it spans the fracture itself (Fig. 5d). In the example illustrated, a bioresorbable polymer material has been modelled for the intramedullary splint, which has a lower UTS than titanium.

## Discussion

This review critically analyses a cohort of rib fracture patients treated with intramedullary splints. The specific intramedullary splint being assessed relies on a single point of fixation with a bicortical screw. The stiffness of the titanium construct achieves fixation at the fracture site by ‘splinting’ the fracture as it rests on the inner cortex at two further points along its length. However, there is no fixation distally as such. As we have demonstrated in the FEA modelling, this does leave room for movement at the fracture site. This may not necessarily be detrimental in itself as micromovements at fracture sites are known to stimulate osteoblast formation and promote bone healing [10, 14]. Analysis of the patient CT scans at 3 months confirmed that it was likely that micromovements were occurring during healing. Failure of complete healing, with minor distraction noted at the fracture sites, but a healthy amount of callus formation would support this hypothesis.

No fracture site displacement was seen in 31 of the 33 rib fractures fixed with the splints. This is notable because we have previously observed that displacement and overlapping typically occurs in non fixed posterior rib fractures [16]. Thus the intramedullary splints were successful in preventing fracture site macro movement.

Use of the intramedullary splints is a little more technically demanding than the outer cortical plates, despite appearing to be a simple fixation solution. The splints can perforate the cortex fairly easily, exiting the medullary canal and entering either the chest wall muscles or the pleural cavity. For this reason we always confirm intramedullary placement of the splint with image intensifier in the operating room prior to completing the case. In the latter part of our experience we would cut off the distal tip of the splint which is only 1 mm in diameter, leaving a wider tip to the splint which seemed less prone to perforating the cortex. The splint can also cause distraction at the fracture site as it is being reamed through the medullary canal of the distal fragment of rib. It is important to prevent this with graspers and keep the fractured ends of the rib in close contact as much as possible.

Use of the Synthes titanium splint for multiple rib fracture fixation could be considered an off label use of the product. Presumably when there are multiple ribs fractured, the displacement forces are greater because there are fewer intact ribs splinting the chest wall. In this analysis we noted no hardware failures, but failure of the rib/splint construct was noted in two patients. Interestingly both patients noted the onset of increased pain immediately after sneezing. Again the splint itself remained intact, but in both patients the superior cortex fractured. Another patient demonstrated partial asymptomatic perforation of the superior cortex of the rib by the distal end of the splint in two consecutive ribs. This mode of failure (superior cortical fracturing) was noted in a bench top study of the titanium intramedullary splints [11]. In that study it was noted that the fractures commenced at the original fracture site and spread along both the superior and inferior cortex.

In another benchtop study, the performance of the titanium rib splints was compared to Kirschner wires [15]. The titanium rib splints performed better than the Kirschner wires with the Kirschner wires demonstrating poor rotational stability resulting in slippage and progressive collapse at the fracture site. The rectangular cross section of the Synthes titanium splints avoid this instability. The study also highlighted the lesser stiffness of the titanium alloy (elastic moduli of 110GPa) compared to the stainless steel of the Kirschner wires (elastic moduli of 220 GPa). The lower construct stiffness of the titanium splints does not indicate lower strength. In contrast, the peak stresses at the points of contact between bone and splint can be reduced by a more elastic implant. As we demonstrated in the FEA modelling, the stainless steel intramedullary construct led to excessive stresses at the bone /implant interface during simulated cough level forces, although it demonstrated no likelihood of splint failure in itself. In contrast, the bioresorbable polymer intramedullary splint with a much lower UTS demonstrated no tendency to excessive stress at the rib/implant interface, but did demonstrate an increased likelihood of splint failure at the fracture site. The properties of a titanium splint fall between those of stainless steel and bioresorbable polymer.

Unfortunately we did not do FEA modelling of the specific titanium splint used clinically in this series. However, the FEA modelling performed does provide us with useful information on the likely displacement occurring at the posterior fracture site as well as the sites of excessive stress on both the rib and an intramedullary splint used to fix the fracture.

We have not compared our results with intramedullary splints to cortical plates in this series as we apply them in different situations. The cortical plates are fairly straightforward to apply to anterior and lateral rib fractures. In the past we had avoided fixation of posterior rib fractures as we noted an increased incidence of hardware failure in this area when we exclusively used absorbable polymer plates [4]. We then moved to titanium plates and have not noted any hardware failures since then. However, these plates are very difficult to apply under the scapula as the bicortical screws must be placed perpendicular to the plate. The use of 90 degree drills and screwdrivers will address this issue but they are not yet widely available. As such we moved to using the MatrixRib intramedullary splints for the difficult to access rib fractures (almost invariably posterior fractures) which has given us this unique opportunity to assess their performance.

Comparison of intramedullary fixation to cortical plate has been performed in a prospective randomised controlled trial of midshaft clavicular fractures [16]. In that study the time to union was equivalent between the two groups. However, residual shortening was significantly greater in the cortical plating group, which the authors attributed to plate bending seen in that group. However, failure to adequately fixate the distal fragment of a fracture could also lead to distraction at the fracture site and apparent lengthening, or failure of union.

The intramedullary splint does offer some theoretical advantages over outer cortical plates. They achieve rib fixation with less hardware than traditional outer cortical plates which require multiple screws to fix the plate to bone. The splint requires only one screw and the majority of the hardware is within the intramedullary canal. This could reduce potential complications such as palpable hardware in thin patients. In this cohort of patients we also utilised this fixation option to reduce the risk of hardware impinging on the moving scapula.

There are currently no prostheses that have been specifically designed for fixing posterior rib fractures. The posterior rib has a different anatomy to the lateral and anterior rib, with thicker cortical bone and a rounder cross section. The posterior rib also follows a tighter radius of curvature than at any other position along the length of the rib. Thus the design of a posterior rib fixation device requires some modifications to the currently available rib fixation prostheses. We have previously demonstrated that the forces acting on posterior rib fractures are greater than on other parts of the rib and that overlap and deformity is a common outcome of the non-fixed posterior rib fracture [9, 16].

## Conclusions

This study is a retrospective descriptive review of a clinical case series and is designed to be hypothesis generating only. Although we noted delayed healing on the follow up CTs, functional recovery in these patients appeared to be comparable to that in patients fixed with outer cortical plates in our centre. Thus the impact of intramedullary rib fixation with a single point of fixation on fracture healing may be only academic. Rib fixation is a rapidly evolving field. After decades of experimental or last resort ‘bail out’ surgery and variable results, there is finally level one evidence emerging showing the benefits of rib fixation. Thus critical analysis of our results as we proceed is crucial to ensure that we continue to offer the best possible surgical solutions for these patients. Use of intramedullary fixation is appealing because it lends itself to minimally invasive access, and may minimise the potential complications of palpable metalwork in thin patients. There are no other rib specific intramedullary splints on the market that we are aware of which is disappointing as this method of fixation offers potential benefits over cortical plates. Further analysis of outcomes with intramedullary splints is warranted as well as further development of similar rib fixation prostheses.
